# A Specialized Odor Memory Buffer in Primary Olfactory Cortex

**DOI:** 10.1371/journal.pone.0004965

**Published:** 2009-03-23

**Authors:** Christina Zelano, Jessica Montag, Rehan Khan, Noam Sobel

**Affiliations:** 1 Department of Neurobiology, Weizmann Institute of Science, Rehovot, Israel; 2 Department of Psychology, University of Wisconsin, Madison, Wisconsin, United States of America; The Rockefeller University, United States of America

## Abstract

**Background:**

The neural substrates of olfactory working memory are unknown. We addressed the questions of whether olfactory working memory involves a verbal representation of the odor, or a sensory image of the odor, or both, and the location of the neural substrates of these processes.

**Methodology/Principal Findings:**

We used functional magnetic resonance imaging to measure activity in the brains of subjects who were remembering either nameable or unnameable odorants. We found a double dissociation whereby remembering nameable odorants was reflected in sustained activity in prefrontal language areas, and remembering unnameable odorants was reflected in sustained activity in primary olfactory cortex.

**Conclusions/Significance:**

These findings suggest a novel dedicated mechanism in primary olfactory cortex, where odor information is maintained in temporary storage to subserve ongoing tasks.

## Introduction

Working memory is a system that holds information in temporary storage during the planning and execution of a task [1]. The prevalent model of working memory holds that this information may be stored in verbal coordinates within a “phonological loop”, in spatial coordinates within a “visuospatial sketchpad” [2], [3], as well as in a third multimodal component termed the “episodic buffer” that combines information from multiple sources [4]. Imaging studies have associated these mechanisms with networks primarily (but not only) within the prefrontal and posterior parietal cortices for the phonological loop and visuospatial sketchpad [5]–[8]. It is unclear, however, how olfactory working memory is represented within this framework. In other words, when one maintains an odor in working memory, what is one remembering, and where?

Several psychophysical studies have suggested that one is remembering a verbal representation of the odor [9]–[13]. Consistent with this, imaging studies of olfactory working memory have revealed prefrontal activity that may indeed reflect involvement of the phonological loop in this process [14].

By contrast, other psychophysical studies suggested working memory for odors entails non-verbal sensory representations of odor *per se* [15]–[18]. Such olfactory sensory representations, by definition, are unlikely to be represented within a phonological loop or visuospatial sketchpad, and may therefore represent a yet-to-be identified olfactory equivalent. Considering the associational properties of primary olfactory cortex [19]–[22], and its involvement in memory processes other than working memory [23], [24], this neural substrate presents a prime candidate for such an olfactory working memory store.

Finally, olfactory information may be held in working memory within a combined network of both verbal and sensory representations [15], [25], where the relative contribution of each mechanism may be a reflection of the nameability of the odor.

**Figure 1:**
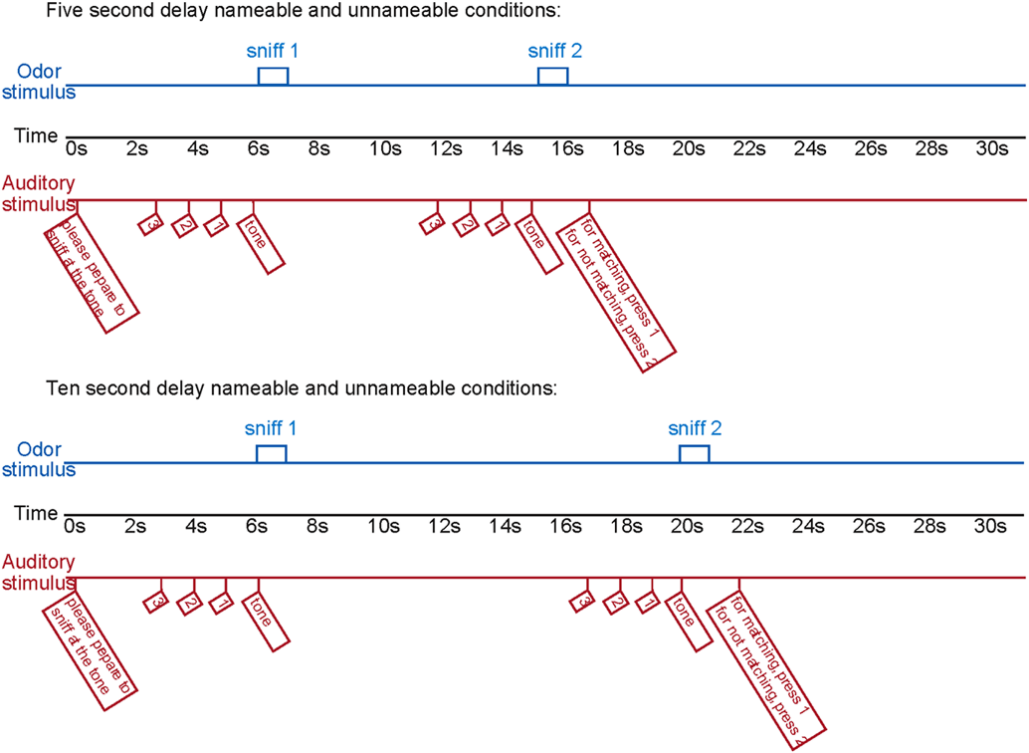
Experimental design. In the scanner, subjects performed an olfactory delayed-match-to-sample memory task where they were presented with two sequential odorants and then asked whether the two odorants were the same or different. The odorants were presented either 5 or 10 seconds apart, and were either nameable or unnameable smells (more specifically, the task instructions were presented 5 or 10 seconds apart, so that sniffs were technically 9 and 14 seconds apart). All subjects received the same odorants, but those odorants differed across subjects in terms of their nameability (see figure S2). The figure shows a schematic of a trial that a participant would experience in the scanner. The blue lines show the olfactometer output, the black line shows time elapsed, and the red lines show the auditory stimulus that the subjects heard through headphones throughout the experiment.

**Figure 2:**
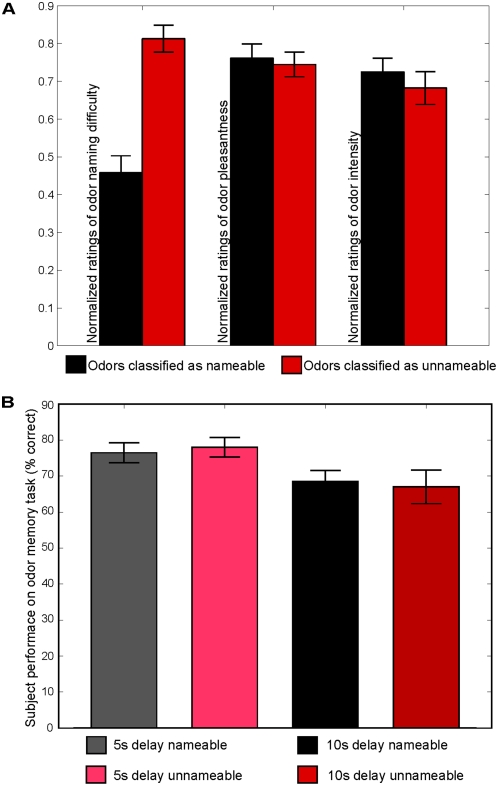
Behavioral results. A. Psychophysical ratings of odorant nameability, pleasantness and intensity. Odorants classified as unnameable were rated as significantly more difficult to name than odors classified as nameable. However, subjects rated both classifications of odorants to be equal in pleasantness and intensity. B. Subjects performance on the delayed match to sample task. Subjects performed better in the conditions with a 5 second delay between odor presentations than the conditions with a 10 second delay between odor presentations. There was no difference in performance across odor subcategories.

To compare these alternative hypotheses we first scanned 10 healthy participants during an olfactory delayed match to sample task (figure 1) wherein they remembered odorants that were either easy to name (from here on *nameable*) or hard to name (from here on *unnameable*) (figure 2A). The task consisted of two sniffs spaced either 5 or 10 seconds apart. The two sniffs, that were the same 50% of the time, consisted of either two nameable odorants, two unnameable odorants, or one nameable and one unnameable odorant. After the second sniff, subjects pressed a button to indicate whether they judged the two odorants as the same or different. In other words, the task demanded that subjects remember a nameable or unnameable odor for either 5 or 10 seconds.

## Results

### Behavior

**Figure 3:**
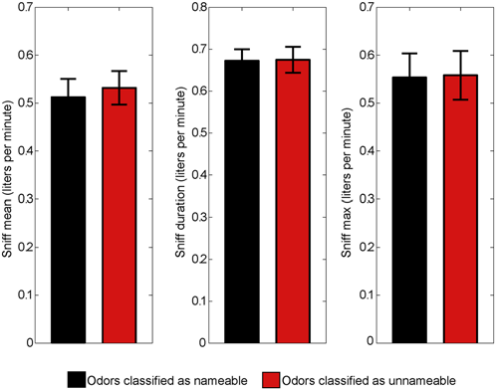
Sniff size across conditions. Participants sniffed equally during nameable and unnameable conditions. The left panel shows the mean sniff, in liters per minute, for nameable and unnameable conditions. The center panel shows sniff duration, in liters per minute, for nameable and unnameable conditions. The right panel shows sniff maximum, in liters per minute, for nameable and unnameable conditions.

Odorants classified as unnameable and nameable were rated as equally intense (t(9) = 0.76, p<.45) and pleasant (t(9) = 0.38, p<.7) yet significantly more difficult to name (t(9) = 9.6, p<.0001) (figure 2a). Subjects' performance on the delayed match to sample task was better for the five second delay conditions than for the ten second delay conditions (F(1,9) = 7.04, p<.02), but did not differ between the nameable and unnameable conditions (F(1,9) = 3.2, p<.11) (figure 2b). Sniffs of nameable odorants did not differ in duration (F(1,9) = 0.08, p<.7853), max flow (F(1,9) = 2.14, p<.1745) or mean flow (F(1,9) = 2.15, p<.1769) from sniffs of unnameable odorants (figure 3). Upon completion of all scans, each subject answered a multiple choice survey questionnaire regarding his/her strategy of remembering the odorants they could or could not name (figure S1). Subjects reported that when they could easily name the odorant, they remembered the word describing it. However, when the odorant was difficult to name, subjects attempted to hold the smell in mind during the delay.

### Increased activity for unnameable vs. nameable odorants in primary olfactory cortex

**Figure 4:**
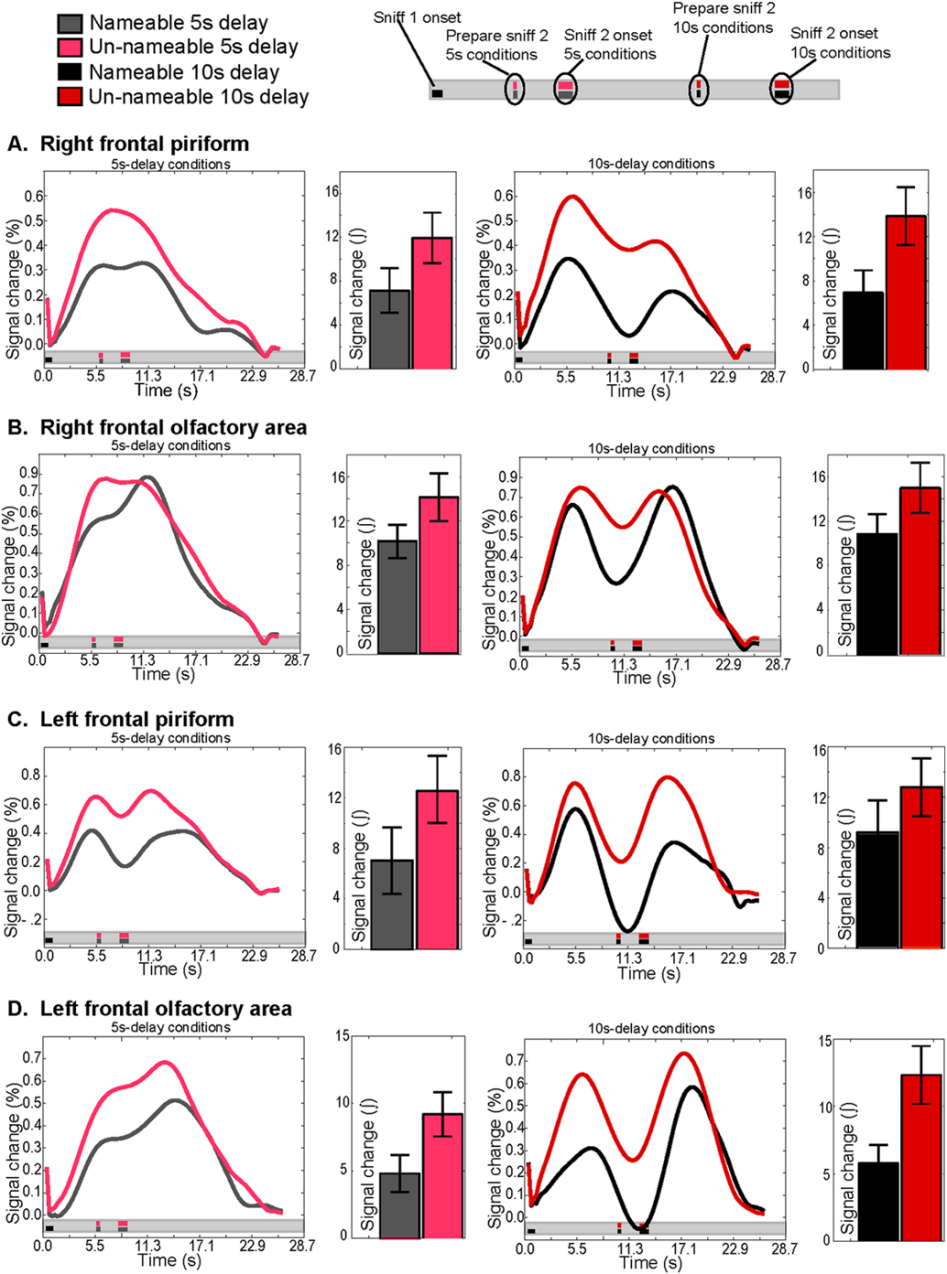
Activity in piriform cortex in response to sniff-one. Each row of the figure shows the deconvolved time course of activity in each region. In each row, the left panel shows the nameable and unnameable 5-second-delay conditions while the right panel shows the nameable and unnameable 10-second-delay-conditions. To the right of each panel is the binned response of each condition. Bar graphs depict the integral under the curve in response to sniff-one of the delayed match to sample task. Error bars represent the standard error. In all subregions shown in the figure, smelling unnameable odorants elicited more activity than nameable odorants. A. Activity in right frontal piriform cortex. B. Activity in right frontal olfactory area. C. Activity in left frontal piriform cortex. D. Activity in left frontal olfactory area.

To determine whether nameable and unnameable odorants were processed differently in primary olfactory cortex, we outlined regions of interest (ROIs) in three primary olfactory subregions: temporal piriform cortex (pirT), frontal piriform cortex (pirF) and the olfactory tubercle (olfA). These regions were selected based on past findings of heterogeneity in response across these subdivisions [26]–[28]. We examined the response in those regions to the first sniff of the delayed match to sample task, and compared its magnitude in response to nameable vs. unnameable odorants (figure 4A–D).

In left and right pirF, there was an effect of odorant nameability (left: F(1,9) = 5.9, p<.03 ; right: F(1,9) = 6.9, p<.03), and no effect of delay time (left: F(1,9) = .07, p<.8 ; right: F(1,9) = .59, p<.45) (figure 4A and 4C). Specifically, in both left and right pirF, the blood oxygen level dependant (BOLD) response to the first sniff was significantly greater in response to smelling unnameable odorants over nameable odorants (left: t(9) = 2.39, p<.02 ; right: t(9) = 2.9, p<.009).

In left and right olfA, there was also an effect of odorant nameability (left: F(1,9) = 20, p<.001 ; right: F(1,9) = 6, p<.03) and no effect of delay time (left: F(1,9) = 3.2, p<.1 ; right: F(1.9) = 3.8, p<.08) (figure 4B and 4D). In left olfA, the BOLD response to the first sniff was greater in response to smelling unnameable odorants as compared to nameable odorants (left: t(9) = 4.6, p<.0001), and a similar trend was evident in right olfA (right: t(9) = 1.85, p<.07).

In right and left pirT, there was no effect of nameability (right: F(1,9) = 1.17, p<.3 ; left: F(1,9) = 1.7, p<.2) or delay time (right: F(1,9) = 3.23, p<.1 ; left: F(1,9) = 0.06, p<.8) in the response to the first sniff.

These results indicated that nameable and unnameable odorants were represented differently in primary olfactory cortex. Specifically, smelling unnameable odorants resulted in more activity in primary olfactory cortex than smelling nameable odorants. This conclusion is strengthened by our design where the same odorants were nameable for some subjects and unnameable for others (figure S2). In other words, the differences in activity likely reflected genuine differences in nameability, rather than some other olfactory trait associated with one group of odorants versus another.

### Remembering unnameable odorants was reflected in sustained activity in primary olfactory cortex

**Figure 5:**
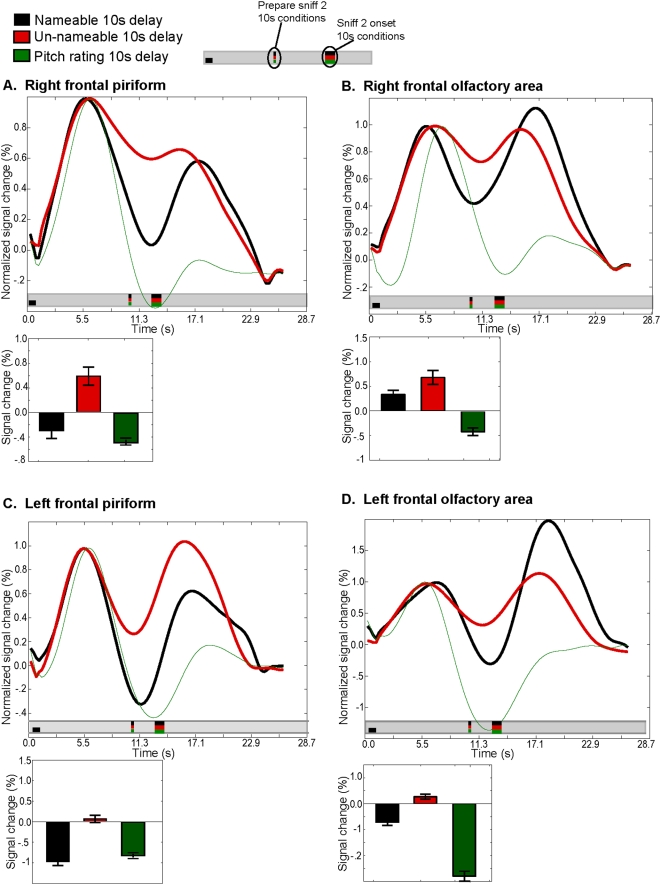
Memory-related activity in piriform cortex. Each panel shows the time course of activity that has been normalized such that the maximum response to sniff-one is equal to 1. This enabled comparisons of activity levels during the delay period. Only the nameable (in black) and unnameable (in red) 10-second-delay conditions are shown along with an additional control condition in which subjects remembered auditory pitches (in green) for 10 seconds. Bar graphs depict the minimum activity level from 6-seconds after the first sniff to the time that the subject was instructed to prepare for the second sniff. This provides a measure of how well activity levels were sustained during the delay (note that because the bar graphs in this figure depict the minimal value, the bar may reveal a negative value even when the mean was positive, e.g., black line in panel A). A. Memory-related activity in right frontal piriform cortex. B. Memory-related activity in right frontal olfactory area. C. Memory-related activity in left frontal piriform cortex. D. Memory-related activity in left frontal olfactory area. In all subregions, the level of activity during the delay period was greater when subjects were remembering unnameable odorants as compared to nameable odorants. In the olfactory area, activity levels were hierarchically organized in the following manner: unnameable>nameable>auditory pitch. In frontal piriform cortex, remembering unnameable odors elicited more activity than nameable odors, but remembering nameable odors and auditory pitches both elicited a similar level of activity during the delay (unnameable>nameable = auditory pitch).

Next, to ask whether sustained activity due to remembering nameable and unnameable odorants was dissociable in primary olfactory cortex, we compared levels of sustained activity during the delay period of the delayed match to sample task (figure 5A–D, figure S3A). Given that the initial response to nameable and unnameable odorants differed in primary olfactory cortex, we normalized the response to the first sniff so that it was the same for nameable and unnameable odorants, allowing for a meaningful comparison between the two odorant categories during the delay (see methods). Because of the lower reliably in dissociating responses from two sniffs taken 5 seconds apart (due to the temporal resolution of fMRI), we restricted this analysis to include only the 10-second delay conditions of the delayed match to sample task.

In right and left pirF, maintenance of unnameable odorants resulted in significantly more sustained activity than maintenance of nameable odorants (right: t(9) = 3.6, p<.001 ; left: t(9) = 3.3, p<.004) (figure 5A and 5C).

Similarly, in right and left olfA, maintenance of unnameable odorants resulted in significantly more sustained activity than maintenance of nameable odorants (right: t(9) = 3, p<.008 ; left: t(9) = 3, p<.008) (figure 5B and 5D).

Additionally, in right pirT, maintenance of unnameable odorants resulted in significantly more sustained activity than maintenance of nameable odorants (t(9) = 3.2, p<.003). In left pirT, however, there was no difference between activity levels during maintenance of nameable and unnameable odorants (p>.05).

### Sustained activity in primary olfactory cortex was specific to olfactory memory

Primary olfactory cortex is situated near the medial junction of the frontal and temporal lobes, proximal to the entorhinal cortex and the hippocampus, areas that are predominantly associated with memory function [29]–[31]. To ask whether the memory-related activity observed in primary olfactory cortex was in fact olfaction-specific, and not a reflection of spatial blurring from nearby structures involved in generalized memory function, we conducted a control experiment in which subjects remembered the pitch of an auditory tone that was presented along with an odorant (thin green lines in figure 5A–D).

In right and left pirF, remembering unnameable odorants resulted in significantly higher levels of sustained activity than remembering auditory tones (unnameable>auditory: right: t(23) = 4.83, p<.0001; left: t(23) = 4.4, p<.0001), but remembering nameable odorants resulted in similar levels of sustained activity as remembering auditory tones (nameable = auditory: right: t(23) = 1.6, p<.12; left: t(23) = 0.74, p<.46) (figure 5A and 5C).

In right and left olfA, remembering both nameable and unnameable odorants resulted in higher levels of sustained activity than remembering auditory tones (LEFT: unnameable>auditory: t(23) = 4.1, p<.0004 ; nameable>auditory: t(23) = 2.9, p<.008; RIGHT: unnameable>auditory: t(23) = 4.8, p<.0001; nameable>auditory: t(23) = 3.1, p<.003) (figure 5B and 5D).

These results confirmed that the memory-related activity we observed in primary olfactory cortex was in fact specific to remembering olfactory stimuli.

### Remembering nameable odorants was reflected in sustained activity in cortical language areas

Given that unnameable odorants were maintained in primary olfactory cortex to a greater degree than nameable odorants, we questioned whether the latter may be preferentially held in different loci. Because working memory for words and other semantic information results in sustained BOLD activity in language areas [32]–[35], we hypothesized that nameable odorants might be preferentially maintained there. To explore this possibility, we outlined ROIs in opercular, orbital and triangular inferior frontal gyrus, and examined the BOLD signal in these regions. We analyzed the time series in these ROIs identically to the time series analysis conducted in primary olfactory cortex. Because anatomical coverage of one participants' brain did not include inferior frontal gyrus, that particular subject was excluded from this analysis.

**Figure 6:**
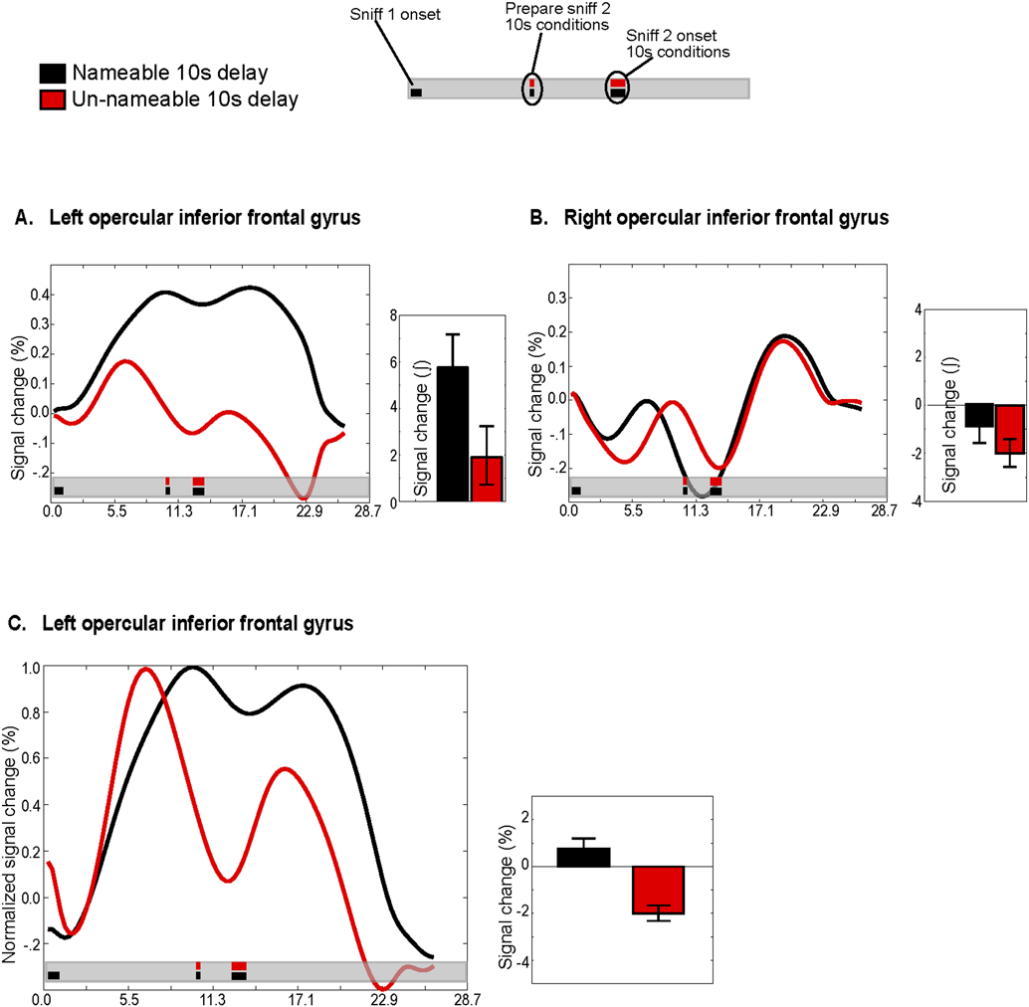
Activity in inferior frontal gyrus. A. The deconvolved time course of activity in left inferior frontal gyrus area. Although we analyzed data from opercular, orbital and triangular inferior frontal gyri, all data shown in the figure are from the opercular region. The smaller panels show the binned response of the 10-second delay nameable and unnameable odorant conditions. Bar graphs depict the integral under the curve in response to sniff-one of the delayed match to sample task. Error bars represent the standard error. Smelling nameable odorants elicited more activity than unnameable odorants. B. The deconvolved time course of activity in right inferior frontal gyrus area is shown as in *A*. On the right side, there was no difference between conditions. C. Memory-related activity in left inferior frontal gyrus. Shown is the time course of activity that has been analyzed identically to the data shown in figure 3. The level of activity during the delay period was greater when subjects were remembering nameable odorants as compared to unnameable odorants.

First, we examined the response to the first sniff of the delayed match to sample task and compared its magnitude in response to nameable vs. unnameable odorants (figure 6A).

In left opercular and orbital inferior frontal gyrus, the observed activity pattern was opposite to the pattern observed in primary olfactory cortex. Again, there was an effect of odorant nameability (opercular: F(1,8) = 12.3, p<.008 ; orbital: F(1,8) = 13, p<.006), and no effect of delay time (opercular: F(1,8) = 2.5, p<.15 ; orbital: F(1,8) = 1.12, p<.3) (figure 6A). Specifically, in contrast to the results in primary olfactory cortex, the BOLD response to the first sniff was significantly greater in response to smelling nameable odorants as compared to unnameable odorants (opercular: t(8) = 2.7, p<.01 ; orbital: t(8) = 2.6 p<.02 ). Consistent with the lateralized nature of language processing in the brain, there was no effect of nameability in the right hemisphere (F(1,8) = 0.38, p<.55) (figure 6B).

Next, to compare levels of sustained activity when remembering nameable and unnameable odorants, we normalized the response to the first sniff, as we did with the time series from primary olfactory cortex (figure 6C, figure S3B). In left opercular, orbital and triangular inferior gyri, maintenance of nameable odorants resulted in significantly more sustained activity than maintenance of unnameable odorants (opercular: t(8) = 3.5, p<.003; orbital: t(8) = 3.1, p<.006; triangular: t(8) = 3.3, p<.005).

In summary, we found that primary olfactory cortex preferentially maintained unnameable odorants, and inferior frontal gyrus maintained nameable odorants. This double dissociation was further evidenced by a region (language vs. olfactory) by odor (nameable vs. unnameable) interaction (F(1,34) = 32, p<.001).

## Discussion

In this study we set out to identify the neural substrates of olfactory working memory. We found that when an odorant was easily named, its maintenance in working memory was reflected in sustained activity preferentially in the area of inferior frontal gyrus. This is consistent with maintenance of this information within a phonological loop. In contrast, the key novel aspect of our results was that when an odor was hard to name, its maintenance in working memory was reflected in sustained activity preferentially in the area of frontal piriform cortex. In other words, olfactory working memory was distributed in a manner that reflected odorant nameability.

One might ask whether the increased activity in primary olfactory cortex during maintenance of unnameable odors was due to differences in task difficulty. This is unlikely for several reasons. Firstly, participants performed equally well when remembering nameable and unnameable odors, suggesting that the difficulty of the task was comparable across conditions (as seen in figure 2, average performance was between 65% and 75% correct, ruling out any ceiling effect). Secondly, if task difficulty were driving the activity pattern observed, we would expect to see a correlation between BOLD activity and naming difficulty within the nameable and unnameable conditions separately. In other words, we would expect that odorants within the nameable condition would elicit less activity depending on how easy they were to name, and odorants within the unnameable condition would elicit more activity depending on how difficult they were to name. On the other hand, if task difficulty were not driving the activity pattern, we would expect all nameable odorants to elicit a similar level of activity and all unnameable odorants to elicit a similar level of activity. To address this, we tested for correlation between BOLD activity and ratings of nameability within each condition. We found no correlation (all p>0.1). Finally, it is important to keep in mind that the task here was not to name the odors, but rather to remember the odors. For all of these reasons combined, it is not likely that differences in task difficulty were driving the activity pattern observed.

Another possible question is whether the activity pattern can be explained by attention or novelty. Unnameable odorants are inherently more novel than nameable odorants. To account for this, we made certain that all participants were exposed to all odorants before going into the scanner (when they rated the pleasantness, intensity and nameability of all odorants). Further accounting for novelty affects, participants repeatedly smelled all odorants while in the scanner because of the nature of the task. Finally, as stated before, subjects were not attempting to identify the odorants but rather to remember them.

Our results indicated that odorants were processed differently as a function of nameability. One may ask at what stage of processing was this difference manifested? Or in other words, when does the olfactory system “decide” whether an odorant is namable? Unfortunately, the fMRI methodology we have used here does not enable us to address questions in the sub-second time domain. Furthermore, one can assume that secondary olfactory cortical regions, such as the orbitofrontal cortex, may have also been involved in the process of olfactory working memory. However, our acquisition and analysis were optemized for primary olfactory cortex. In several cases we lacked functional coverage of secondary olfactory cortices, and thus cannot here determine or characterize their involvement in olfactory working memory.

It is important to stress that the dissociation between nameable and unnameable odorants was relative. That is, there was more sustained activity in olfA for both nameable and unnameable odors than for auditory tones, suggesting the brain makes use of both these working-memory stores in order to maintain an active odor image. Even when an odor is not easily named, the olfactory system may use a form of verbal labeling, perhaps reflecting some more generalized stimulus attributes or categorizations. That said, the difference across all regions in relative sustained activity as a function of nameability was robust. In other words, there was clear preferential sustained activity in piriform cortex for unnameable odorants, and in inferior frontal gyrus for nameable odorants. Finally in this respect, one should keep in mind that because we normalized the response to the first sniff in order to compare the response during the delay, we did not compare absolute measurements of sustained activity during the delay period.

The finding of sustained activity reflecting working memory for odors in primary olfactory cortex was the key finding in this study. Similar to a visuospatial sketchpad where the brain briefly stores images, this function can be likened to an olfactory *flacon* (the French word for *flask*, often used to describe a vessel for perfume), where the brain briefly stores odors. Should this novel mechanism now be added as a third and equally independent mechanism of working memory together with the visuospatial sketchpad and phonological loop [36]? Two key features of the visuospatial sketchpad and phonological loop are indeed reflected in the olfactory flacon: First, a notable aspect of both the phonological loop and the visuospatial sketchpad is that rather than simple maintenance of sensory information, they both reflect information from a number of different sources that are not dependent upon the sensors themselves. Hence one can form a visual image of a verbally described object, and a phonological representation of a printed word. Similarly, recent evidence suggests this is true of olfactory cortex as well, where activity is increased following visual presentation of objects previously associated with odors [19], following reading a word such as *cinnamon* that represents an odor [37], and following efforts to imagine olfactory stimuli [38]. Furthermore, a second important aspect of the visuospatial sketchpad and phonological loop is that the representation within these mechanisms can be actively manipulated. Again, a similar situation is evident in olfactory cortex, where activity patterns reflect the imagined hedonic value of an odor. Imagining an unpleasant odor induces activity similar to that induced by smelling unpleasant odors, and imagining pleasant odors induces activity similar to the induced by smelling pleasant odors [39]. In other words, activity in this region was actively manipulated.

Finally, early models of brain organization highlighted the separation between mechanisms of memory storage and mechanisms of sensation. Our findings of neural representations for olfactory working memory in primary olfactory cortex, combined with findings such as neural representations of tactile working memory in primary somatosensory cortex of monkeys [40] and humans [41], together blur the distinction between sensory perception and working memory [42], and suggest extending the consideration of working memory beyond the framework of a “phonological loop”, “visuospatial sketchpad”, and “episodic buffer” alone.

## Materials and Methods

### Subjects

Twelve subjects (six women and six men, ranging in age from 18 years to 38 years) participated in the main experiment (remembering nameable and unnameable odors). Data from two subjects was deemed unusable: one for excessive motion, and the other due to the subject falling asleep and not performing the task. Fifteen additional subjects (four women and eleven men, ranging in age from 19 years to 31 years) participated in the control experiment (remembering auditory pitch ratings). All subjects gave written informed consent to procedures approved by the UC Berkeley Committee for the Protection of Human Subjects.

### Odorants and olfactometry

Odorants were delivered by a computer-controlled air-dilution olfactometer that also provided an ongoing real-time measurement and recording of airflow in the nostrils [43]. This olfactometer switches between odorant presence and absence in less than 2 ms, with no non-olfactory cues as to the alteration. The odorants used in experiment 1 were octanoic acid, decyl-alcohol, chocolate oil, benzyl-alcohol, orange oil, L-carvone, benzaldehyde, phenyl-ethy-alcohol, garlic, octanol and various mixtures of these compounds. All were presented at suprathreshold concentrations. The odorant used in experiment 2 was amyl acetate.

### Experimental design

In an event-related design, subjects performed a delayed-match-to-sample task while in the scanner. Each trial consisted of the presentation of two odorants separated by either a five or ten second delay. After each trial, the subject was instructed to press 1 if the odorants matched, or 2 if they did not. To ask whether the task involved the active memory of a word (ie the name of the odor) or an odor percept, we chose odors that were either nameable or un-nameable for each subject. To do this, we conducted psychophysical experiments on each subject individually to determine which odors were easy and difficult for that subject to name. Subjects came into the lab before going into the scanner, sampled each odor and then filled out a rating form for each sample. On the rating form, subjects were asked to come up with a name for the odor, and then to rate how difficult it was to come up with that name on a sliding scale. Subjects also rated the pleasantness and intensity of the sample. After this process, to determine which odors fell into which category (nameable or unnameable), each subject's nameability ratings were normalized, to create a personal nameability scale for each subject ranging from 0 to 1. We then took odors falling below 0.5 to be nameable and odors falling above 0.5 to be unnameable for each subject. This way, nameable and unnameable odors were tailored to each subject (figure S2), and differed across subjects, thereby reducing the possibility that other characteristics of the odors themselves (ie pleasantness and intensity) were responsible for the pattern of activity we observed. Thus, we had four conditions: nameable 5 or 10 second delay, and unnameable 5 or 10 second delay. Each of the four trial types was presented 20 times across five 592-second long functional scans, using an inter-trial-interval of 34.5 or 39.5 seconds, depending on the delay time (the exact number of presentations of each trial varied slightly between subjects, as subjects may have had differing numbers of odors the fell above and below 0.5 on their particular scale. However, this number did not vary by more than 1 for any given subject, ie, no subject had a deviation from equal numbers that was more than 1).

In the second experiment, each trial of an event-related design began with an auditory primer instructing the subject to prepare to sniff at the tone. At the tone, the olfactometer administered an odorant to the subject. Twenty-four trials were presented in which subjects were instructed to remember the pitch of the auditory tone indicating the subject to sniff. Tones were increasing, decreasing, or flat in pitch. After a 10 second delay, subjects were asked to press 1 if the tone was decreasing, 2 if the tone was flat, and 3 if the tone was increasing in pitch.

### Imaging parameters

All the raw MR data are publicly available at http://www.weizmann.ac.il/neurobiology/worg/materials.html. The experiment was conducted on a 4T Varian Inova magnet. A custom-built full-head receive coil was used for signal reception. A T2* sensitive echo plainer sequence was employed with parameters of TR = 500 ms, TE = 28 ms, flip angle = 20°, phi = 270. The functional in-plane resolution was 3 mm and the through-plane resolution was 3.5 mm. Twenty 3.5-mm thick slices were acquired at a coronal plane, so as to cover primary olfactory cortex.

### Imaging analysis

Data were analyzed using MrVista (http://white.stanford.edu/software/). Following initial coregistration and motion estimation and correction, one subject showed evidence of head movement beyond correction and was excluded from further analysis.

Piriform cortex is defined cytoarchitectually, and its exact borders cannot be delineated based on the MR image alone. Here we combined a structural and functional restriction in order to define the region of interest. We first outlined the expected subregions of piriform based on an atlas that is particularly detailed in this respect [44] (figure S4A) . In the atlas, piriform cortex is divided into three parts: a temporal portion (pirT), a frontal portion (pirF), and another frontal portion that lies medial to pirF and is called the olfactory area (olfA). OlfA likely includes the olfactory tubercle and other small portions of primary olfactory cortex. We then functionally restricted these regions to only voxels that responded hemodynamically to a separate “localizer” scan, in which subjects were simply instructed to sniff at the tone every thirty seconds for 7 minutes (p<0.01). Each sniff contained a different odorant, and the analysis contrasted sniffing the odorant versus an passive baseline. Thus, the localizer activates both sniff-activated regions and odorant-activated regions. We then analyzed the time series in each of these ROIs. The time series corresponding to each trial (an interval extending from 10 s before sniff onset to 45 s after it) was smoothed (convolved with a Gaussian of FWHM = 2 s), detrended, normalized by subtracting the average response from t = 10 s before odorant onset up to 1 s before the time of odorant onset, for the whole time series (the baseline response before sniff) so that all time series had comparable baselines, and then fitted to the mean response. Because of the importance of temporal resolution in this study, we included two additional analysis techniques. Firstly, each TR was divided into four equal subsections in time, and each sniff was determined to have occurred closest to the beginning of one of the subsections. The time series were then shifted for each trial for each subject to reflect this more accurate measure of exactly when the sniff occurred. Secondly, each time series was deconvolved with an impulse response function. The fMRI signal is heavily filtered by its inherent delay, resulting in the blurring of temporally evolving events. Deconvolution acts as a deblurring method [45]. Because individual variations in the shape of the hemodynamic response function indicate that the best method for deconvolution of BOLD time series obtained from several subjects is to determine an individual impulse response function for each subject [45], we used each subject's mean response to the localizer scan as his/her impulse response function. Finally, we calculated the average of all correct trials of a particular condition to derive an average time series for that ROI.

To draw ROIs in the opercular, orbital and triangular inferior frontal gyrus, we used the same atlas used to outline piriform ROIs (figure S4B). Because the slices acquired for one particular subject's brain were not located sufficiently anterior to cover inferior frontal gyrus, this subject had to be excluded from this analysis. The time series in these ROIs were analyzed identically to those in piriform cortex with one important difference. Because using an odor-defined-localizer would not be logical when looking for language-related activity, we chose to outline these ROIs very conservatively, and not further functionally restrict them. This added the risk of weakening effects by the addition of voxels with other more varied response profiles, yet rendered more robust any effects we did find in this region.

The fMRI response to the first sniff of the delayed match to sample task was defined as the area under the hemodynamic response curves in the window 3 to 6 s after odorant presentation. It is important to note here that in certain cases (as seen in figure 6, for example), the area under the BOLD signal can be negative. While a positively valued signal indicates an increase in tissue oxygen concentration, a negatively valued signal simply indicates a decrease in tissue oxygen concentration. The precise meaning of an overall decrease in tissue oxygen concentration, and therefore a negative BOLD response, has been the topic of numerous studies. The most recent of these found a correlation between negative BOLD and GABA concentration, indicating that negative BOLD is correlated to neural inhibition [46]. For each ROI, we performed a random-effects model three-way ANOVA with subject as the blocking variable and odor nameability and delay time as the grouping variables. Because we were looking at the response to sniff-1 before the subject knew whether the delay time would be 5 or 10 seconds, we expected no effect of delay time in any ROIs. We looked for main effects as well as second-order interactions between these factors.

The fMRI response during the delay period of the delayed-match-to-sample-task was defined as the minimum of the hemodynamic response curves in the window 7 to 10 seconds after odorant presentation, when the time series were normalized according to the maximum value of the response to sniff-1. To allow for a meaningful comparison between conditions during the delay period, it was essential that we normalize the response during sniff-1, as it differed between conditions. For example, had we not normalized the sniff-1 response, and then found significantly more activity in the unnameable condition during the delay, we would not know if that significant difference was due to the initial increase in activity for unnameable odors in response to sniff-1, or rather reflected an actual significant difference between the conditions during the delay. In other words, the initial differences between conditions could act as a kind of condition-specific baseline shift when looking separately at the delay time window, thereby obscuring the meaning of any differences between conditions during that delay time window. Because we were unable to resolve the response during the delay period and the response to the second sniff in the 5-second delay conditions, this analysis was restricted to only the 10-second delay conditions. We then analyzed data from experiment two in an identical way, and compared the conditions of interest: nameable vs. unnamable, nameable vs. auditory tones and unnameable vs. auditory tones. This amounts to 3 comparisons per region.

## Supporting Information

Figure S1 (0.49 MB TIF)

Figure S2 (0.01 MB TIF)

Figure S3 (0.45 MB TIF)

Figure S4 (1.11 MB TIF)
