# Supplementary figures and images for: A Specialized Odor Memory Buffer in Primary Olfactory Cortex
Source: PLoS One. 2009 Mar 23;4(3):e4965. doi: 10.1371/journal.pone.0004965 (PMC2654926; doi:10.1371/journal.pone.0004965)

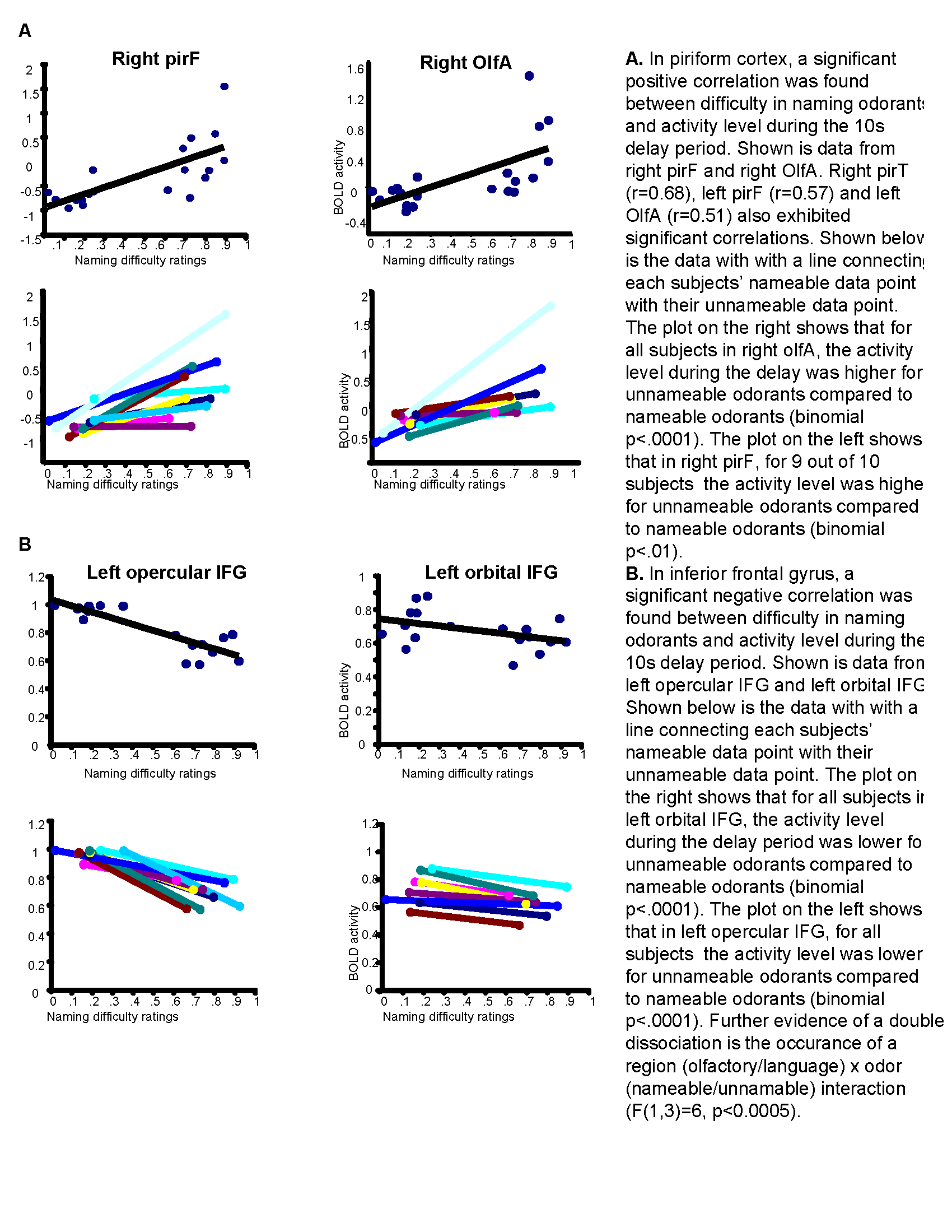

Supplement: Figure S3 — (0.45 MB TIF) [file pone.0004965.s003.tif]

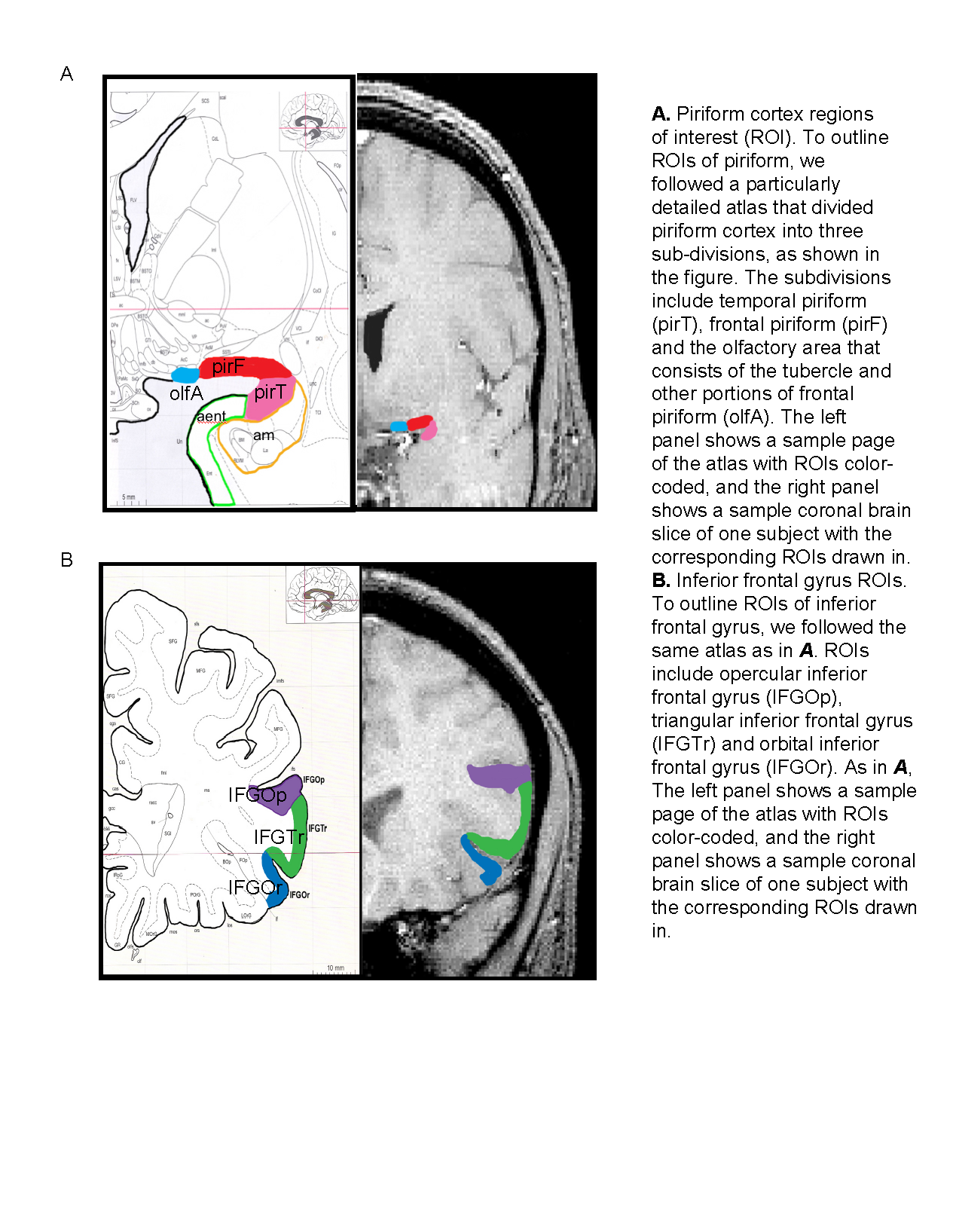

Supplement: Figure S4 — (1.11 MB TIF) [file pone.0004965.s004.tif]
